# Dynamic Compressed HRRP Generation for Random Stepped-Frequency Radar Based on Complex-Valued Fast Sequential Homotopy

**DOI:** 10.3390/s140508283

**Published:** 2014-05-08

**Authors:** Peng You, Zhen Liu, Hongqiang Wang, Xizhang Wei, Xiang Li

**Affiliations:** School of Electronic Science and Engineering, National University of Defense Technology, Changsha 410073, China; E-Mails: ypnudt@126.com (P.Y.); oliverwhq@vip.tom.com (H.W.); liwerer@nudt.edu.cn (X.W.); lixiang01@vip.sina.com (X.L.)

**Keywords:** high resolution range profile (HRRP), random stepped-frequency, dynamic compressed sensing, fast sequential homotopy, recursive sparse recovery, group sparse

## Abstract

Compressed sensing has been applied to achieve high resolution range profiles (HRRPs) using a stepped-frequency radar. In this new scheme, much fewer pulses are required to recover the target's strong scattering centers, which can greatly reduce the coherent processing interval (CPI) and improve the anti-jamming capability. For practical applications, however, the required number of pulses is difficult to determine in advance and any reduction of the transmitted pulses is attractive. In this paper, a dynamic compressed sensing strategy for HRRP generation is proposed, in which the estimated HRRP is updated with sequentially transmitted and received pulses until the proper stopping rules are satisfied. To efficiently implement the sequential update, a complex-valued fast sequential homotopy (CV-FSH) algorithm is developed based on group sparse recovery. This algorithm performs as an efficient recursive procedure of sparse recovery, thus avoiding solving a new optimization problem from scratch. Furthermore, the proper stopping rules are presented according to the special characteristics of HRRP. Therefore, the optimal number of pulses required in each CPI can be sought adapting to the echo signal. The results using simulated and real data show the effectiveness of the proposed approach and demonstrate that the established dynamic strategy is more suitable for uncooperative targets.

## 1. Introduction

Radar can be used to achieve high resolution images of targets. High range resolution profiles (HRRPs) are easier to obtain compared to their high dimensional counterparts and can be used in many applications including target classification and recognition, *etc.* Linear stepped-frequency radar (LSFR) is an effective way to synthesize high resolution range profiles of targets, which can be implemented by a frequency-agile transmitter with a narrowband receiver [1]. Thus, LSFR can be applied to complement the imaging capability for narrowband radar systems. In order to synthesize a large bandwidth for LSFR with narrowband pulses, one must make an appropriate tradeoff between pulse repetition frequency (PRF) and coherent processing interval (CPI) from a traditional view. On the one hand, in the cases of fixed low and median PRFs, a large number of pulses should be transmitted to cover the whole frequency band, thus resulting in long observation times. However, modern radars often have other missions besides imaging, such as detecting and tracking, which will limit the observation time for each target. Furthermore, for long CPIs, both radial- and micro-motions of the target will seriously distort the pulse coherence and thus degrade the performance of HRRP generation with LSFR. On the other hand, when the CPI is fixed, high PRF is usually required for LSFR to achieve enough coherent pulses, which will induce range ambiguity and blind zones for farther targets.

One effective way to achieve large bandwidth with low PRF during short CPI is to employ the sparse stepped-frequency radar (SSFR) [2], in which there are far fewer pulses in the train due to the frequency band vacancy. For SSFR, there are mainly two sparse patterns, *i.e.*, the periodic pattern and the random pattern, both of which will degrade the HRRP quality if we directly apply traditional pulse compression techniques. For the periodic pattern combined with Fourier transform, grating lobes will appear to cause aliasing for HRRPs, and for the random pattern, the correlation processing suffers from high sidelobe pedestals, which will decrease the peak to sidelobe ratio.

In order to reduce the grating lobes and sidelobe pedestal caused by missing frequency bands in SSFR, high quality HRRPs are achieved in [2,3] by applying the sequence CLEAN method, which is essentially a simple sparse-seeking approach and can be viewed as an implementation of matching pursuit [4]. More recently, owing to the advances in optimal recovery from sparsity constraints [5], especially the emergence of compressed sensing (CS) theory [6,7], exploiting sparsity in high resolution processing for SSFR has been attracting growing attention. For example, the sparsity-driven HRRP synthesis based on Bayesian CS (BCS) is proposed in [8] for the SSFR with periodic patterns. In [9,10] the CS theory is also applied to form HRRPs of moving targets using the SSFR with random pattern. However, these are only pilot studies and focus on finding the sparse HRRP for a given set of echoes. For practical applications, the required number of pulses is difficult to determine in advance under various scenarios and the reduction of the transmitted pulses is attractive. A meaningful solution is that we transmit the HRRP pulses and update the estimated HRRP with sequentially received pulses until the proper stopping rules are satisfied. In this case, the existing static recovery procedures always suffer from low efficiency due to having to solve a new inverse problem from scratch when the echo pulses are available sequentially.

In this paper, we propose a novel algorithm to quickly update the estimated HRRP without solving a new optimization problem from scratch as well as establishing the proper stopping rules. Due to the linearity of frequency, LSFR as well as SSFR with periodic pattern have the “diagonal ridge” ambiguity function, which suffers from serious delay-Doppler coupling [11]. Therefore, herein we only focus on SSFR with random patterns, which can also be called random stepped-frequency radar (RSFR) [12]. Besides delay-Doppler decoupling, excellent resistance to range ambiguity and electronic countermeasures can also be achieved by the randomly transmitted frequencies in RSFR, which even satisfy the request of randomness for the restricted isometry property (RIP) condition [13] and guarantee that HRRP can be recovered exactly with high probability [10]. The main contributions of this paper are thus in the following three aspects:

(1) A dynamic compressed sensing strategy for HRRP generation is provided. In this strategy, the estimated HRRP is updated with sequentially transmitted and received pulses. Furthermore, the number of required pulses within each CPI is determined adaptively for attaining an acceptable HRRP in various scenarios, e.g., different targets and/or different target aspects.
(2) A complex-valued fast sequential homotopy (CV-FSH) algorithm is developed to efficiently implement sequential update. This approach is an extension to the fast sequential homotopy (FSH) proposed in [14], which has been proved more effective for sequential solution in the noisy settings than its counterparts [14]. It should be noted that the extension is not straightforward. We first reformulate the complex-valued sparse recovery as a group sparse recovery model. Then the fast sequential update for group sparse recovery is completed by resorting to the homotopy technique.
(3) Proper stopping rules for the dynamic approach are given based on the special characteristics of HRRP. With these rules, we can seek the optimal number of pulses required in each CPI adapting to the echo signal. In the experiments, simulated and real data are used to test the proposed approach. The results show its effectiveness for both stationary and moving targets.

The paper is organized as follows: Section 2 describes the signal model of RSFR and the sequential compressed sensing (SCS) based dynamic strategy for HRRP synthesis is derived. In Section 3, the CV-FSH algorithm is developed to implement fast sequential processing of SCS and some proper stopping rules are given for the dynamic approach in Section 4. Section 5 presents some results achieved by using both simulated and chamber measured data, which validate the effectiveness of the proposed algorithm. The last section gives the conclusion. In this paper, the operations of transposition and conjugate transposition are denoted by superscripts *T* and *H*, respectively. Operators Re(·) and Im(·) select the real part and imaginary part of the argument, respectively. ‖***x***‖*_p_* denotes the *l_p_*-norm operation of ***x***, and |·| denotes the absolute operation.

## 2. Dynamic High Range Resolution Profile (HRRP) Synthesis in Random Stepped-Frequency Radar (RSFR)

### 2.1. Echo Signal Model of RSFR

**Figure 1.**
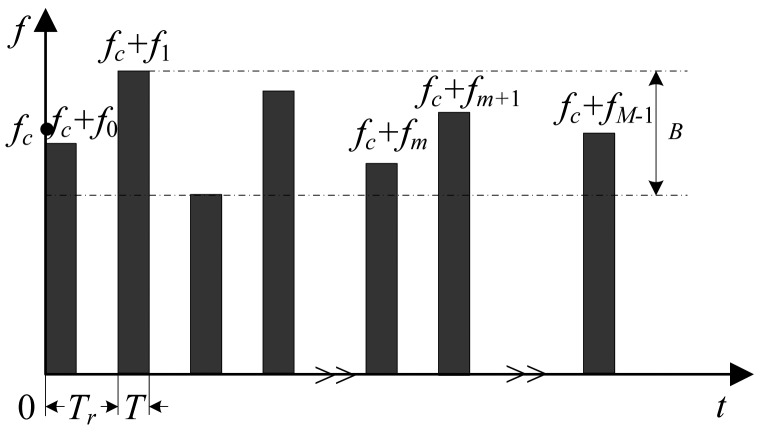
Pulse train signal model of Random stepped-frequency radar (RSFR).

As shown in Figure 1, the pulse train signal used in RSFR is composed of *M* pulses, where *M* is to be determined according to various scenarios. The carrier frequency of the *m*th pulse is *F_m_* = *f_c_* + *f_m_*, where *f_c_* is the fundamental carrier frequency and *f_m_* = *C_m_*Δ*f* is randomly distributed in the bandwidth *B* with a basic frequency step Δ*f*. *C_m_* is an integer randomly selected from {0,1,…,*N* −1}, *N* = *B*/Δ*f*. The pulse repetition interval (PRI) is *T_r_* and the pulse width is *T*.

Assume that the extended rigid target has *K* scattering centers projected on the radar line of sight (LOS) and that the aspect of the target with respect to radar remains unchanged during the coherent processing interval (CPI) for HRRP synthesis. The intensity of the *k*th scattering center is *A_k_* and its initial distance apart from the radar is *R*_0_*_k_*. When the target moves with a constant radial velocity of *v* (positive when towards the radar), then, after down-conversion and low-pass filtering, the echo signals can be represented as:

(1) $$s_{rm}(t)=\sum_{k=1}^{K}A_{k}^{\prime }\text{rect}\left(\frac{t-mT_{r}-T/2-t_{k}}{T}\right)\exp(-j2\pi F_{m}t_{k})$$

where $\mathrm{rect}\left(\frac{t}{T}\right)=\{\begin{array}{cc} 1, & -T/2\leq t\leq T/2\\ 0, & else \end{array}$, *m* = 0,1,…,*M* − 1, $A_{k}^{\prime }$ is the echo envelop and $t_{k}=\frac{2(R_{0k}-vt)}{c}=t_{0k}-\frac{2vt}{c}$, *c* is the velocity of light. Sampling the signal at $t_{m}=mT_{r}+t_{0}^{s}$ ( $t_{0}^{s}=2R_{0}^{s}/c$, and $R_{0}^{s}$ represents the sampling distance nearest the target), then the output signal can be expressed as:

(2) $$s_{r}[m]=\sum_{k=1}^{K}A_{k}^{\prime }\exp\left[-j2\pi F_{m}(t_{0k}-2vt_{m}/c)\right]$$

Assume that the unambiguous delay (*i.e.*, the PRI) *T_r_* is divided into *N* (*i.e.*, the number of range cells, which is larger than *M*) points as $T_{n}=t_{0}^{s}+n\Delta t$with Δ*t* = 1/*B* and *n* = 1,…,*N* − 1, and the velocity domain of interest *v_u_* is divided into *L* points as *V_l_* = *v_c_* + *l*Δ*v*, where *v_c_* is the initial target radial velocity and *l* = 0,1, *L*−1. Then, by considering noisy cases, Equation (2) can be expressed as:

(3) $${\text{s}}_{r}={\text{U}}_{l0}\text{a}+\text{n}$$

where ***U****_l_*_0_ is an *M* × *N* random matrix, whose elements are *U_l_*_0_0[*m*,*n*] = exp(−*j*2π*F_m_T_n_*)exp(*j*4π*F_m_T_m_V_l_*_0_/*c*) with *V_l_*_0_ = *v*. The vector ***a*** = [*a*_0_,*a*_1_,…,*a_N_*_−1_]*^T^* just represents HRRP, in which there are about *K* non-zero elements. ***n*** represents white zero-mean measurement noise.

### 2.2. SCS-Based HRRP Synthesis for RSFR

Obviously, Equation (3) can be regarded as a typical underdetermined linear system, and the randomness of carrier frequencies ensures that the equivalent observation matrix is a randomly sampled sub-Fourier matrix, which could meet the RIP condition [15]. Moreover, if the signal-to-noise ratio (SNR) is sufficiently high, and the number of main scattering centers projected on the radar line of sight is sufficiently smaller than the number of range cells to guarantee the following sparse condition [13]:

(4) $$M\geq O\left(K\cdot {\left(\log N\right)}^{4}\right)$$

then Equation (3) can be regarded as a typical CS model in the complex-valued domain and the HRRP can be obtained by solving the following basis pursuit denoising (BPDN) problem:

(5) $$\text{â}=\arg\underset{\text{a}}{\min}\frac{1}{2}{\left\|s_{r}-{\text{U}}_{l0}\text{a}\right\|}_{2}^{2}+\lambda {\left\|\text{a}\right\|}_{1}(5)$$

where λ is a regularization parameter.

**Figure 2.**
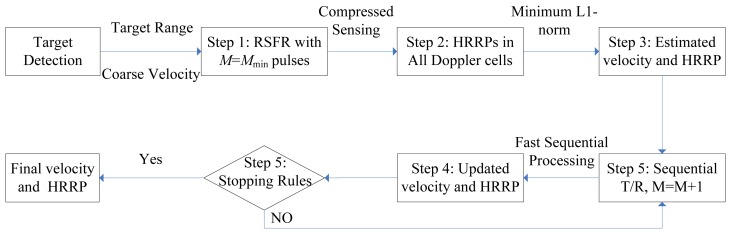
Flow chart of the dynamic HRRP generation processing for RSFR.

In practice, however, the number of main scattering centers is always changing for different targets or the same target with various attitudes, thus, the number of pulses is difficult to choose *a priori*. As the observations are available in sequence for RSFR, herein we can assume that the number of pulses in each CPI is bounded in a proper scope and then the echo signal can be more accurately modeled as an SCS problem [16]. Unlike traditional scheme which is performed after all the echo signals are obtained, here we can generate the HRRPs sequentially with echo pulses from the minimum number to the maximum number. In this situation, the interval time between each pulse can be exploited for fast sequential processing. The dynamic algorithm for HRRP synthesis as well as motion compensation is shown in Figure 2 and listed as the following steps.


**Algorithm 1** Dynamic HRRP synthesis and motion compensation

Step 1Initialize the number of pulses as the minimum number, *i.e.*, *M* = *M_min_*. Assume the unambiguous velocity achieved directly from tracking system or by resolution of any Doppler ambiguity (depending on the tracking scheme) is *v̄* and the velocity measurement accuracy is ± *δv*, then the velocity domain of interest can be set as *v_u_* = [*v̄* − *δv*, *v̄* + *δv*], the initial velocity is:
(6)$$v_{c}=\bar{v}-\delta v$$and the number of velocity cells is *L* = 2*δv*/Δ*v*, where Δ*v* is the velocity step size.Step 2The HRRP in the *l*th velocity cell ${\text{â}}_{M}^{l}$ is figured out by resolving the following BPDN problem:
(7)$${\text{â}}_{M}^{l}=\arg\underset{{\text{a}}_{M}^{l}}{\min}\frac{1}{2}{\left\|{\text{s}}_{rM}-{\text{U}}_{M}^{l}{\text{a}}_{M}^{l}\right\|}_{2}^{2}+\lambda_{M}^{l}{\left\|{\text{a}}_{M}^{l}\right\|}_{1}$$where ${\text{U}}_{M}^{l}$ is an *M* × *N* random matrix with elements $U_{M}^{l}[m,n]=\exp(-j2\pi F_{m}T_{n})\exp(j4\pi F_{m}t_{m}V_{l}/c)$ and $\lambda_{M}^{l}$ is the corresponding regularization parameter.Step 3The minimum *l*_1_-norm criterion is applied for motion compensation by calculating the *l*_1_-norm of every ${\text{â}}_{M}^{l}$and searching the minimum to get:
(8)$$({\text{â}}_{M},{\text{î}}_{M})=\arg\underset{{\text{â}}_{M}^{l},l}{\min}{\left\|{\text{â}}_{M}^{l}\right\|}_{1}$$where ***â**_M_* is the estimated HRRP for the *M* pulses and *î_M_* is the estimation of the corresponding velocity cell.Step 4When the (*M* + 1) the sample of echo signal is obtained, we have ${\text{s}}_{rM+1}=\left[\begin{array}{c} {\text{s}}_{rM}\\ {\text{s}}_{r}[M+1] \end{array}\right]$.Set ${\text{U}}_{M+1}^{l}=\left[\begin{array}{c} {\text{U}}_{M}^{l}\\ {\text{u}}_{M+1}^{l} \end{array}\right]$with ${\text{u}}_{M+1}^{l}=\left[u_{M+1}^{l}[0],\ldots ,u_{M+1}^{l}[N-1]\right]$and $u_{M+1}^{l}[n]=\exp(-j2\pi F_{M+1}T_{n})\exp(j4\pi F_{M+1}t_{M+1}V_{l}/c)$. Take ${\text{â}}_{M}^{l}$, ***â**_M_* and *î_M_* as the “warm-starts” and re-estimate ${\text{â}}_{M+1}^{l}$, ***â****_M_*_+1_ and *î_M_*_+1_ by resolving the following two problems:
(9)$${\text{â}}_{M+1}^{l}=\arg\underset{{\text{a}}_{M+1}^{l}}{\min}\frac{1}{2}{\left\|{\text{s}}_{rM+1}-{\text{U}}_{M+1}^{l}{\text{a}}_{M+1}^{l}\right\|}_{2}^{2}+\lambda_{M+1}^{l}{\left\|{\text{a}}_{M+1}^{l}\right\|}_{1}(9)$$
(10)$$\left({\text{â}}_{M+1},{\text{î}}_{M+1})=\arg\underset{{\text{â}}_{M+1}^{l},l}{\min}{\left\|{\text{â}}_{M+1}^{l}\right\|}_{1}(10\right)$$Step 5If the estimated HRRP and velocity cell for the *M*+1 pulses are sufficiently similar to these for the *M* pulses or the number of pulses reaches the maximum number, *i.e.*, *M* + 1 =*M*_max_, then with high probability the ***â****_M_*_+1_ obtained in Step 4 is the HRRP we need and the estimation corresponding to *î_M_*_+1_ is just the target velocity *v̂_M_*_+1_ = *V_l_*_^_*_M_*_+1_, else enter a new transmission and reception (T/R), and set *M* = *M* + 1, ***s****_rM_* = ***s****_rM_*_+1_, ${\text{U}}_{M}^{l}={\text{U}}_{M+1}^{l}$, ***â****_M_* = ***â****_M_*_+1_, *î_M_* = *î_M_*_+1_, then go to Step 4.


The fast sequential processing algorithm for update is developed in Section 3. In Section 4, some stopping rules are discussed and the proper stopping rules are given.

## 3. Fast Sequential Processing of SCS

Although the HRRPs of the target can be obtained well with the dynamic algorithm proposed in the last section, there are still two important issues from practical consideration. One is how to avoid solving a new optimization problem from scratch when the new echo pulses are available sequentially. The other is how to design proper stopping rules for attaining acceptable results in various scenarios, which can determine the minimum number of pulses required and make the CPI the shortest. Therefore, in this section we first assume that the BPDN (7) has already been solved by using the homotopy technique, which is computationally efficient and has a simple regularization parameter setting [17], and then the FSH approach [14] is extended to resolve (9) more efficiently. In FSH, homotopy algorithm is modified to update the solution for BPDN when a new measurement in the real-valued domain is added to the system sequentially. In Subsection 3.1, the complex-valued sparse recovery is transformed into a real-valued group sparse recovery model. The quick update of group sparse recovery solution based on the homotopy technique is presented in Subsection 3.2.

### 3.1. Group Sparse Recovery Model for Complex-Valued Sparse Recovery

In order to make the deduction simpler, we rewrite the abstract signal model in the complex-valued domain as well as the BPDN problem as:

(11) $$s_{r}=\text{Ua}+\text{n}$$

(12) $$\text{â}=\arg\underset{\text{a}}{\min}\frac{1}{2}{\left\|\text{Ua}-{\text{s}}_{r}\right\|}_{2}^{2}+\lambda {\left\|\text{a}\right\|}_{1}$$

where ***U*** ∈ ℂ*^M^*^×^*^N^* is the equivalent sensing matrix, ***s****_r_*, ***n*** ∈ ℂ*^M^*^×1^ are vectors of the measurements and complex noise, respectively, ***a*** ∈ ℂ*^N^*^×1^ is a sparse vector and λ is a regularization parameter. As shown in [18], in order to use the sparse recovery techniques in the real-valued domain, the complex-valued model should be firstly transferred to the real-valued one by defining:

(13) A=[Re(U),−Im(U)Im(U),Re(U)]

(14) y=[Re(sr)Im(sr)],x=[Re(a)Im(a)],e=[Re(n)Im(n)]

From Equation (14), it can be seen that ***x*** is group sparse since Re(***a***) and Im(***a***) show the same sparse support, *i.e.*, they are nonzero with the same indices, which would improve the recover performance if properly used [19]. Actually ***x*** has *N* groups corresponding to *N* complex range resolution cells with their real parts and imaginary parts as group members. By exploiting the group sparse a prior with *l*_2,1_ norm [20], Equations (11) and (12) can be transformed into:

(15) y=Ax+e

(16) $$\hat{\text{x}}=\arg\underset{\text{x}}{\min}\frac{1}{2}{\left\|\text{A}x-\text{y}\right\|}_{2}^{2}+\lambda \sum_{i=1}^{N}\sqrt{x_{i}^{2}+x_{i+N}^{2}}$$

where *x_i_* is the *i*th element of ***x***. With ***x*** estimated by Equation (16), the HRRP ***a*** can be achieved using Equation (14). *N* is the total number of the sparse groups, *i.e.*, the total range resolution cells.

### 3.2. Complex-Valued Fast Sequential Homotopy (CV-FSH) Algorithm

In this Subsection, we will extend the FSH approach to the complex domain. Assume that we have solved Equation (16) to get the estimation ***x***_0_ as well as ***a***_0_ (***a***_0_ = ***x***_0_[1: *N*] + *j* · ***x***_0_ [*N* +1:2*N*]) with our current set of measurements for some given value of λ. Now suppose that we get a new measurement given as $s_{r}^{\prime }={\text{u}}^{\prime }\text{a}+n^{\prime }$, where ***u′*** ∈ ℂ^1×^*^N^* is a row vector and *n′* denotes the complex noise in the new observation. Similarly, the real-valued model can be written as:

(17) [Re(sr′)Im(sr′)]=[Re(u′),−Im(u′)Im(u′),Re(u′)]x+[Re(n′)Im(n′)]

which can also be regarded as adding two real-valued measurements sequentially.

Herein, we consider adding the real part of $s_{r}^{\prime }$.The same way can be used to deal with the imaginary part. By defining w=Re(sr′), ***b*** = Re(***u′***), −IM(***u′***) and *d* = *Re*(*n′*), the system of equations becomes:

(18) $$\left[\begin{array}{c} \text{y}\\ w \end{array}\right]=\left[\begin{array}{c} \text{A}\\ \text{b} \end{array}\right]\text{x}+\left[\begin{array}{c} \text{e}\\ d \end{array}\right]$$

Now we need to solve the following problem for update:

(19) $${\hat{x}}^{\prime }=\arg\underset{x}{\min}\frac{1}{2}\left({\left\|\text{Ax}-\text{y}\right\|}_{2}^{2}+{\left|\text{bx}-w\right|}^{2}\right)+\lambda \sum_{i=1}^{N}\sqrt{x_{i}^{2}+x_{i+N}^{2}}$$

Since the method used here is similar to the one in [14], we provide a description paralleling that of [14]:

(1) Homotopy parameters establishment
  According to the homotopy technique, we need first to introduce continuous homotopy parameters linking the solved optimization program to the new one so that we can trace the solution path by varying the homotopy parameters carefully. From Equations (16) and (19), by introducing the parameter ε, the new measurement is incorporated gradually as:
  (20) $$x^{*}=\arg\underset{x}{\min}\frac{1}{2}\left({\left\|\text{Ax}-\text{y}\right\|}_{2}^{2}+ɛ{\left|\text{bx}-w\right|}^{2}\right)+\lambda \sum_{i=1}^{N}\sqrt{x_{i}^{2}+x_{i+N}^{2}}(20)$$
  While *ε* increases from 0 to 1, the solved problem in Equation (16) changes to the new one in Equation (19). By resorting to the homotopy technique, we can trace the path of solutions, *i.e.*, from ***x̂*** to ***x̂′***, as we slowly vary the parameter ε.
(2) Optimality Conditions Determination
  By differentiating the cost function in Equation (20) with respect to ***x***, the optimality conditions for any solution x* to Equation (20) can be formulated as:
  (21) $${\text{A}}_{\Gamma }^{T}(\text{A}{\text{x}}^{*}-\text{y})+ɛ{\text{b}}_{\Gamma }^{T}(\text{b}{\text{x}}^{*}-w)=-\lambda \text{D}({\text{x}}_{\Gamma }^{*})$$
  (22) $$\left|{\text{A}}_{\Gamma^{C}}^{T}(\text{A}{\text{x}}^{*}-\text{y})+ɛ{\text{b}}_{\Gamma^{C}}^{T}(\text{b}{\text{x}}^{*}-w)\right|<\lambda \left|\text{D}({\text{x}}_{\Gamma^{C}}^{*})\right|$$
  where Γ is the support of the solution x*, *i.e.*, the nonzero or active element index set. Γ*^C^* is the complemental set. (***H***)_Γ_ denotes the new matrix/vector extracted from matrix/vector ***H*** with columns/elements indexed by Γ:
  (23) $$\text{D}({\text{x}}_{\Gamma }^{*})={\left[d_{1},d_{2},\ldots ,d_{\left|\Gamma \right|}\right]}^{T},\;d_{i}=\left\{ \begin{array}{l} x_{\Gamma (i)}^{*}/\sqrt{{\left(x_{\Gamma (i)}^{*}\right)}^{2}+{\left(x_{\Gamma (i)+N}^{*}\right)}^{2}},\;\Gamma (i)\leq N\\ x_{\Gamma (i)}^{*}/\sqrt{{\left(x_{\Gamma (i)}^{*}\right)}^{2}+{\left(x_{\Gamma (i)-N}^{*}\right)}^{2}},\;\Gamma (i)>N \end{array}\right.$$
  where |Γ| is the cardinality of the index set Γ, [·]*^T^* denotes the transpose of a vector and Γ(*i*) denotes the *i*th element of Γ.
  Given the sparse support Γ, Equation (21) is the standard optimality conditions of nonlinear programming. Equation (22) holds since the sparsity exploitation term, the last in Equation (19), is more sensitive to the deviation of inactive elements than the representation error term shown in the bracket, *i.e.*, the inactive elements contribute mainly for the sparsity enhancement and little for the representation error reduction.
(3) Solution Path Tracing
  Assume we are at one of these critical values of ε =ε*_k_* and the solution ***x****_k_* has been achieved. Adding ε with an infinitesimal amount to *ε_k_*′ we can evaluate the corresponding solution ***x****_k_*′ with ∂***x*** = ***x****_k_*′ − ***x****_k_* by using the optimality conditions Equation (21) and first-order Taylor approximation:
  (24) $$\partial \text{x}=\left\{ \begin{array}{l} -({{ɛ}_{k}}^{\prime }-{ɛ}_{k}){\left({\text{A}}_{\Gamma }^{T}{\text{A}}_{\Gamma }+{\text{D}}^{\prime }({\left({\text{x}}_{k}\right)}_{\Gamma })+{{ɛ}_{k}}^{\prime }{\text{b}}_{\Gamma }^{T}{\text{b}}_{\Gamma }\right)}^{-1}{\text{b}}_{\Gamma }^{T}(\text{b}{\text{x}}_{k}-w),\text{ on}\;\Gamma \\ 0,\;\text{otherwise} \end{array}\right.$$
  where:
  (25) $${\text{D}}^{\prime }({\left({\text{x}}_{k}\right)}_{\Gamma })=\text{diag}([d_{1},d_{2},...,d_{\left|\Gamma \right|}]),\;d_{i}=\left\{ \begin{array}{l} {\left({\left(x_{k}\right)}_{\Gamma (i)+N}\right)}^{2}/\sqrt[{3}]{{\left[{\left({\left(x_{k}\right)}_{\Gamma (i)}\right)}^{2}+{\left({\left(x_{k}\right)}_{\Gamma (i)+N}\right)}^{2}\right]}^{2}},\;\Gamma (i)\leq N\\ {\left({\left(x_{k}\right)}_{\Gamma (i)-N}\right)}^{2}/\sqrt[{3}]{{\left[{\left({\left(x_{k}\right)}_{\Gamma (i)}\right)}^{2}+{\left({\left(x_{k}\right)}_{\Gamma (i)+N}\right)}^{2}\right]}^{2}},\;\Gamma (i)>N \end{array}\right.$$

The derivations of Equations (24) and (25) are given in the Appendix A. Through reformulating ∂***x*** = θ*_k_*∂***x***, we can derive the update direction ∂***x*** and the positive step size θ*_k_* as follows using the matrix inversion lemma. The details are presented in the Appendix B:

(26) $$\partial \text{x}=\{\begin{array}{ll} -{\text{U}}^{-1}{\text{b}}_{\Gamma }^{T}(\text{b}x_{k}-w), & \text{on}\;\Gamma \\ 0, & \text{otherwise} \end{array}$$

(27) $$\theta_{k}=\frac{{{ɛ}_{k}}^{\prime }-{ɛ}_{k}}{1+({{ɛ}_{k}}^{\prime }-{ɛ}_{k})u}$$

where $\text{U}={\text{A}}_{\Gamma }^{T}{\text{A}}_{\Gamma }+{\text{D}}^{\prime }({\left({\text{x}}_{k}\right)}_{\Gamma })+{ɛ}_{k}{\text{b}}_{\Gamma }^{T}{\text{b}}_{\Gamma }$, $u={\text{b}}_{\Gamma }{\text{U}}^{-1}{\text{b}}_{\Gamma }^{T}$.

The solution moves away from ***x****_k_* along the direction ∂***x*** as ε increases, until the current support Γ must be modified. That means certain existing element of ***x****_k_* indexed within Γ will shrink to zero or certain one indexed within Γ*^C^* will be active. For the last case, the corresponding inequality in Equation (22) will become an equation. Furthermore, the modification is made according to the required smallest step size.

For the active element shrinking to zero, *i.e.*, turning into the inactive one, the smallest step size is:

(28) $$\theta^{-}=\underset{\gamma \in \Gamma ,\gamma \leq N}{\min}\sqrt{{\left[{\left(\frac{-x_{k}(\gamma )}{\partial x(\gamma )}\right)}_{+}\right]}^{2}+{\left[{\left(\frac{-x_{k}(\gamma +N)}{\partial x(\gamma +N)}\right)}_{+}\right]}^{2}}$$

where γ is the group index. Since *x_k_*_+1_(γ) = *x_k_*(γ) + θ∂*x*(γ) do certainly not shrink toward zero with $\frac{-x_{k}(\gamma )}{\partial x(\gamma )}$ and positive step size, we use (•)_+_ to denote that the minimum is only taken over the group indices with both parts shrinking toward zero simultaneously. Assuming the group γ^−^ needs the smallest step size.

For the inactive element turning into the active one, the required step size is what makes any one of the inequalities in Equation (22) become an equation, which can be written as:

(29) $$\left|{\text{A}}_{\Gamma^{C}}^{T}(\text{A}{{\text{x}}_{k}}^{\prime }-\text{y})+{{ɛ}_{k}}^{\prime }{\text{b}}_{\Gamma^{C}}^{T}(\text{b}{{\text{x}}_{k}}^{\prime }-w)\right|\leq \lambda \left|\text{D}({\left({{\text{x}}_{k}}^{\prime }\right)}_{\Gamma^{C}})\right|$$

where the equation holds for some *j* ∈ Γ*^C^*, which means the inactive element *j* is turning into the active one.

Substituting ***x****_k_′* = ***x****_k_* + θ*_k_*∂***x*** into Equation (29) and setting:

(30) $${\text{p}}_{k}={\text{A}}^{T}(\text{A}{\text{x}}_{k}-\text{y})+{ɛ}_{k}{\text{b}}^{T}(\text{b}{\text{x}}_{k}-w)$$

(31) $${\text{d}}_{k}=({\text{A}}^{T}\text{A}+{ɛ}_{k}{\text{b}}^{T}\text{b})\partial \text{x}+{\text{b}}^{T}(\text{b}{\text{x}}_{k}-w)$$

Then Equation (29) can be rewritten as:

(32) $${\left({\text{p}}_{k}\right)}_{j}+\theta_{k}{\left({\text{d}}_{k}\right)}_{j}\approx \pm \lambda \text{D}({\left({{\text{x}}_{k}}^{\prime }\right)}_{j})=\pm \lambda \text{D}({\left({\text{x}}_{k}\right)}_{j})$$

where the second equation holds since (∂***x***)*_j_* = 0. The first approximation is deduced in Appendix C. It should be noted that Equations (30) and (31) are consistent with Equation (35) in [14]. Thus, the smallest θ*_k_* satisfying Equation (32) is given by:

(33) $$\theta^{+}=\underset{\gamma \in \Gamma^{C},\gamma \leq N}{\min}\sqrt{{\left[\theta (\gamma )\right]}^{2}+{\left[\theta (\gamma +N)\right]}^{2}}$$

where:

(34) $$\theta (\gamma )=\min{\left(\frac{\gamma D_{k}(\gamma )-p_{k}(\gamma )}{d_{k}(\gamma )},\frac{\lambda D_{k}(\gamma )+p_{k}(\gamma )}{-d_{k}(\gamma )}\right)}_{+}$$

(35) $$D_{k}(\gamma )=x_{k}(\gamma )/\sqrt{{\left[x_{k}(\gamma )\right]}^{2}+{\left[x_{k}(\gamma +N)\right]}^{2}}$$

θ(γ) is the smaller step size to achieve Equation (32) for the real part of group γ, with θ(γ + *N*) for imaginary part. Suppose that the group γ^+^ needs the smallest step size.

Thus the step size corresponding to the critical point is:

(36) $$\theta =\text{min}\left(\theta^{+},\theta^{-}\right)$$

If θ = θ^+^, the corresponding inactive element turns into the active one. Otherwise, the corresponding active element turns into the inactive one.

Then, according to Equation (27), the new critical point is:

(37) $${ɛ}_{k+1}={ɛ}_{k}+\frac{\theta }{1-\theta u}$$

and the updated solution is:

(38) $${\text{x}}_{k+1}={\text{x}}_{k}+\theta \partial \text{x}$$

Based on the above idea, CV-FSH can be described by Algorithm 2.


**Algorithm 2** Dynamic update with sequential complex measurements

Step 1Initialize ***x****_k_* as solution ***x***_0_ to Equation (16) with support Γ.Step 2Adding the real part of the measurement.Set *k* = 0, ε_0_ = 0, w=Re(sr′) and ***b*** = [Re(***u′***),−Im(***u′***)].Step 3Iteration:
(1)Compute the update direction ∂***x*** with Equation (26).(2)Compute the step size θ with Equations (28), (33) and (36).(3)Update critical point and the solution with Equations (37) and (38), respectively(4)If ε*_k_*_+ 1_ > 1then $\theta =\frac{1-{ɛ}_{k}}{1+(1-{ɛ}_{k})u}$, ***x****_k_*_+1_ ***x****_k_* + θ∂***x***, ε*_k_*_+ 1_ = 1break (quit the loop);end if(5)If θ = θ^−^Γ←Γ\{γ^−^,γ^−^ + *N*}elseΓ←Γ∪{γ^+^,γ^+^ + *N*}end if(6)*k* ←*k* + 1 until stopping criterion is satisfied.Step 4Adding the imaginary part of the measurement.Set A=[A[Re(U′),−Im(U′)]], y=[yRe(sr′)], w=Im(sr′), ***b*** = [Im(***u′***), Re(***u′***)] ***x***_0_ = ***x****_k_*_+1_, *k* = 0, ε_0_ = 0, and execute the Iteration (Step 3) again.Output: The updated solution *x****^′***.


## 4. Stopping Rules

When the number of pulses is not sufficient, the results of the sparse recovery are inaccurate and will change notably even when only one new measurement is added. Otherwise, the results are accurate and become stable with new added measurements. Therefore, we can define the stopping rules mainly from the one-step agreement of two sequential results.

Obviously, one of the necessary conditions to stop the processing is that the radial velocities are equivalent for the two sequential processing results, *i.e.*, *V_îM_*_+1_ = *V_îM_*, which will guarantee that the motion compensation is well performed. This is because the estimated mean velocity remains nearly unchanged if only one more PRI is added to CPI, which is comparatively much longer. Another stopping criterion is the sufficient similarity of the HRRPs obtained by the two sequential processing. Assume that for a certain instance the minimum number of required pulses is *M*_opt_ which just satisfies the RIP condition, then we can expect that, when *M* < *M*_opt_, the similarity between two adjacent reconstructed HRRPs will be relatively low because of the large error in CS reconstruction, whereas the two adjacent reconstructed HRRPs will be matched very well when *M* > *M*_opt_. Therefore, the value of *M*_opt_ can be estimated by measuring the normalized correlation coefficient between the *M*th and (*M* + 1)th HRRPs, which can be given as [21]:

(39) $$c_{M+1}=\frac{\left|{\text{â}}_{M}^{H}{\text{â}}_{M+1}\right|}{{\left\|{\text{â}}_{M}\right\|}_{2}{\left\|{\text{â}}_{M+1}\right\|}_{2}}$$

If the correlation coefficient is smaller than a certain threshold *c*_th_, the value of *M* must be increased and one more pulse whose carrier has not been used ever should be transmitted accordingly. On the contrary, a correlation coefficient larger than *c*_th_ implies that the HRRP has already been correctly generated and the dynamic processing can be stopped.

Finally, it can be concluded that for the proposed dynamic algorithm, if the number of pulses satisfies the following three conditions simultaneously, the HRRP and estimated velocity of the target can be well obtained with high probability: (1) The number of pulses is among the presetting scope; (2) The estimated velocity is equivalent with the previous one; (3) The normalized correlation coefficient between the current HRRP and the previous one is sufficiently high to approach one.

## 5. Experiments

In this section, the feasibility and performance of the proposed dynamic HRRP generation algorithm are tested on both simulated and chamber measured data. Our simulations are performed in the MATLAB7 environment using a Pentium (R) 4 CPU 3.00 GHz processor with 1 GB of memory.

### 5.1. Effectiveness of Complex-Valued FSH (CV-FSH)

In this subsection, simulations with an ideal scattering center model are carried out to validate the effectiveness of the complex-valued FSH algorithm. The parameters of the transmitted RSFR signal are set as follows: the carrier frequency ranges from 9.5 GHz to 10.5 GHz, *T_r_* =100 μs and *T* = 0.1 μs. It can be calculated that Δ*t* = 1 ns and *N* = 100. Assume that the numbers of targets' scattering centers *K* are set to be 20, 30, respectively and they are located within length 15 m in the LOS. The real parts and imaginary parts of their amplitudes are both randomly distributed **[**img**]**(0,1)^2^. The distance from the target to the radar is 6 km and the target is assumed to be stationary. The minimum and maximum numbers of transmitted pulses are 30 and 90, respectively. When the target has been detected, an RSFR signal is transmitted to obtain the HRRPs of the target under white Gaussian noise, the real parts and imaginary parts of which are both randomly distributed **[**img**]**(0,0.01^2^). For comparison, the complex-valued FSH (CV-FSH) algorithm as well as real-valued FSH (RV-FSH) algorithm and the well-known Convex (CVX) toolbox [22] are applied to 200 Monte Carlo trials. For RV-FSH, complex-valued sparse recovery Equation (11) is transformed into the real-valued one Equation (15). However, RV-FSH exploits the conventional sparse a prior only, compared to CV-FSH imposing group sparse a prior. Thus, RV-FSH is a direct application of the approach proposed by [14].

**Figure 3.**
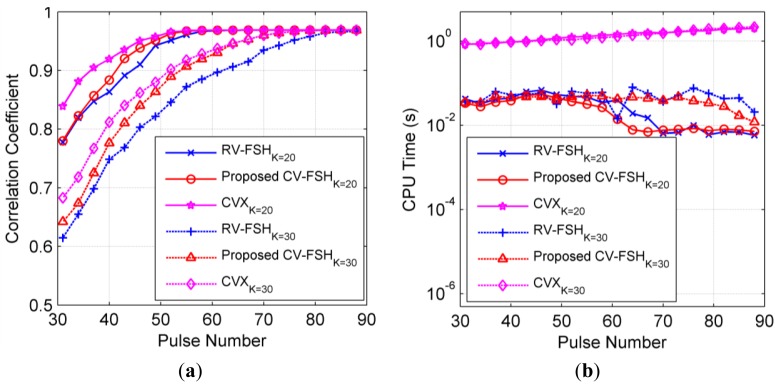
HRRP generation performance of three algorithms with various number of scattering centers. (**a**) Average correlation coefficients between the actual scene and the recovered scenes *versus* number of pulses; and (**b**) Average CPU time required to complete each algorithm *versus* number of pulses.

The average normalized correlation coefficients (Ancc) between the actual scene and the recovered scenes are calculated for each step and the results are shown in Figure 3a, from which it can be seen that with all three algorithms the Ancc rises quickly with the increase of the number of pulses and then tends to a constant approaching one when the number of pulses is large enough. Furthermore, it can also be seen that with the increase of the number of pulses and the decrease of the number of scattering centers, e.g., *M* ≥ 3*K*, the RIP condition is gradually satisfied, which will lead to accurate parameter estimation with the recovery algorithm. For the precise solution algorithm of convex optimization problem Equations (12), (16) and (19) in the CVX toolbox, it performs slightly better than our proposed CV-FSH algorithm, which is a fast pursuit-like method to the update problem (19) and approaches the CVX solutions quickly as the number of pulses increases. RV-FSH performs worst since it imposes a weak prior or loses some information from the information theory perspective.

Figure 3b presents the average CPU time required to complete each algorithm. The curves show that the cost time of the CVX toolbox increases for larger scale of the sensing matrix. However, for the RV-FSH and the CV-FSH algorithms, the cost times almost remain unchanged and even decrease when the results are stable. This is because when the results change slightly toward the true solution, it can provide better and better initialization for faster converging in the updating procedure. In Figure 3b, it can be seen that the CPU times required for RV-FSH and the CV-FSH algorithms begin to reduce notably when the number of pulses is about three times of the number of scattering centers. This phenomenon occurs since the support remains almost unchanged when the number of pulses overpasses certain number and so only few iterations are required to update the solution. CV-FSH achieves the stable point more quickly where the cost time begins to be low and almost unchanged owing to its superior recovery performance.

Therefore, considering both computational complexity and the recovery performance, we observe that the CV-FSH algorithm is more suitable for our problem. It is a great potential to update the solution real-time or quasi-real-time with sequential measurements by resorting to CV-FSH.

**Figure 4.**
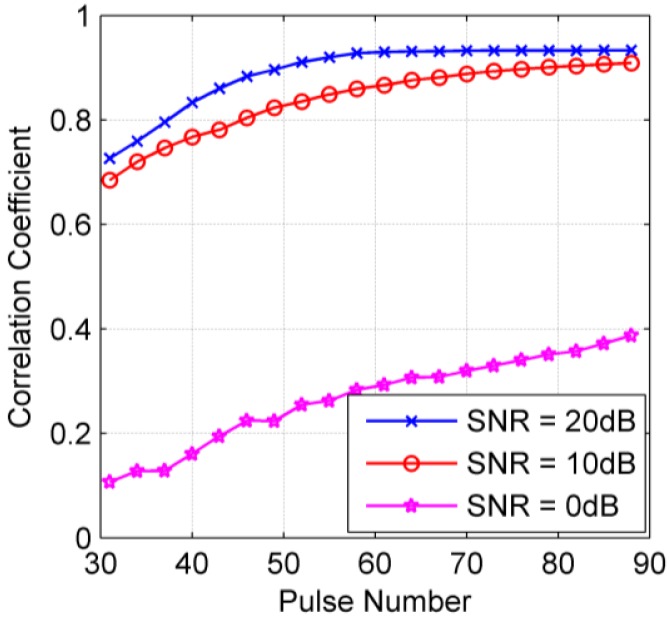
HRRP generation performance with various numbers of pulses under different SNRs.

The CV-FSH algorithm is further analyzed concerning its robustness of HRRP generation. Here we mainly consider how the algorithm performs when adding different white Gaussian noises. By setting the variance of noise as 0.01, 0.1, 1 (corresponding to SNR 20 dB, 10 dB and 0 dB), respectively, 100 Monte Carlo simulations are performed for each case and the Ancc between the actual scene and the recovered scenes are calculated. As shown in Figure 4, as the SNR decreases, the Ancc converges more slowly, *i.e.*, the required number of measurements for exact recovery increases. When the SNR is too low (*i.e.*, SNR = 0 dB), the algorithm is almost ineffective. This phenomenon is similar to the CVX toolbox [22]. Actually, the descending performance of sparse-based algorithms under lower SNR is still an open problem. In the actual radar imaging applications, the required SNR of echo data for imaging is often higher than that for detecting and tracking. Therefore, from the engineering point of view, the stability of the algorithm with SNR higher than 10 dB can achieve most application requirements.

### 5.2. Sequential HRRP Generation of Stationary Target

**Figure 5.**
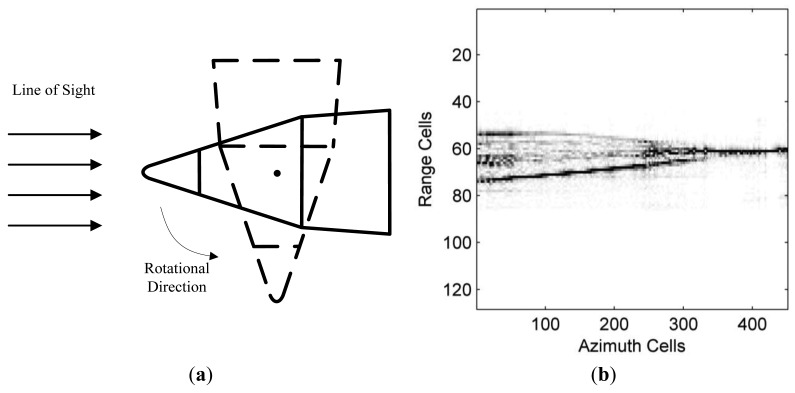
(**a**) Model of the anechoic chamber measured target; and (**b**) Normalized HRRPs for all azimuths.

Before testing the feasibility of Algorithm 1, we first make some experiments to validate the effectiveness of the SCS algorithm for sequential HRRP generation of the stationary target in Figure 5a. The radar for anechoic chamber measuring works in the sweep mode, whose carrier frequencies change from 9 GHz to 11 GHz with an interval of 20 MHz and the center frequency is *f_c_* = 10 GHz. The elevation angle of the target is 0°, and the azimuth angle varies from 0° to 90° (nose direction is 0°) with an interval of 0.2°. By performing 128-point sparse recovery (*i.e.*, the number of range cells is set to be 128) on the 100 samples of the wideband echo signal for each azimuth cell, we can obtain all 450 HRRPs of the target, which are shown in Figure 5b. It should be noted that in the following analysis the HRRP obtained from the whole raw data are called the original HRRP.

As the RSFR is still under development, for convenience, herein we randomly draw out *M* (from 16 to 64) measurements from 100 samples of the LSFR to simulate the echo signal for RSFR. For stationary targets, this is equivalent to the samplings of output signal in RSFR which transmits the *M* pulses at corresponding frequencies. Since the selection of the samples intervening in the SCS approach is done randomly, the repeatability of the proposed method has to be checked. In order to do so, 200 Monte Carlo simulations are performed at two typical azimuths (5° and 45°) to determine the proper number of measurements. In a general way, choosing a larger number of experiments is more reasonable. However, an appropriately small number is preferred for the low computation load. What is more, hundreds or even tens of independent trials are sufficient in the sense that the outcome change slightly when more independent trials are added, which is observed in our simulations. Nevertheless, strict analysis especially mathematical justification about the appropriate number of experiments is still valuable. This open problem deserves further research.

**Figure 6.**
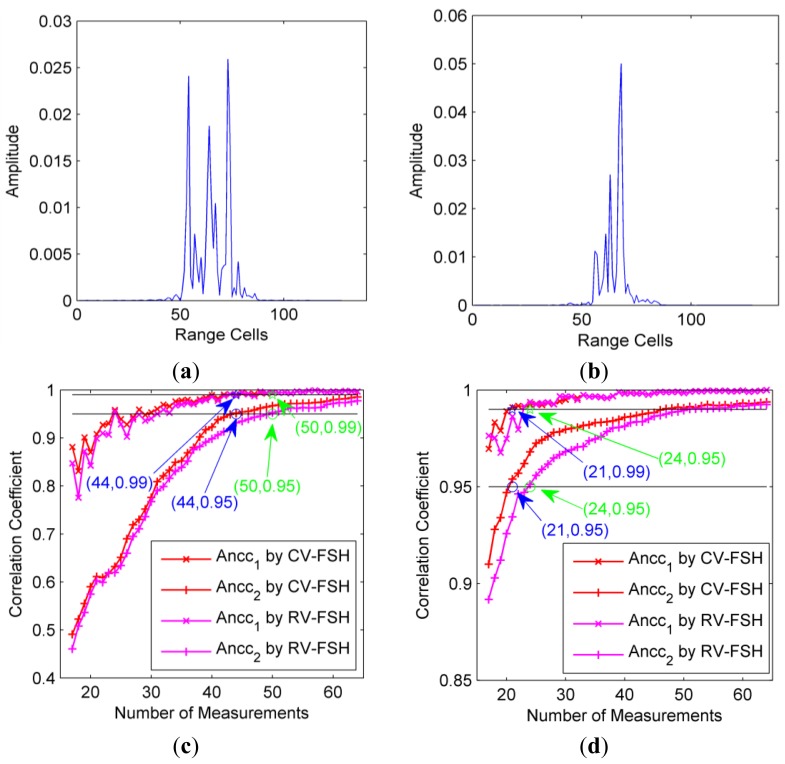
Results of the SCS algorithm for HRRP generation of the stationary target. (**a**) Original HRRP for azimuth 5°; (**b**) Original HRRP for azimuth 45°; (**c**) Ancc_1_ and Ancc_2_ for azimuth 5°; and (**d**) Ancc_1_ and Ancc_2_ for azimuth 45°.

Firstly, the original HRRPs for the two azimuths are given in Figure 6a,b, which obviously show that the number of strong scattering centers for azimuth 45° is smaller than that for azimuth 5°. Due to its much larger computation load, CVX is not appropriate at all to implement the fast sequential update, the core of our dynamic compressive sensing strategy. Thus, the CV-FSH approach is applied to the selected measurements and compared with RV-FSH. Figure 6c and d present the Ancc between two adjacent HRRPs (Ancc_1_) for various numbers of measurements. For comparison, the Ancc between the recovered HRRP and the original HRRP (Ancc_2_) are also given. For CV-FSH, it can be seen that when the HRRP tends to be stable at about 44 measurements for azimuth 5° (*i.e.*, Ancc_2_ is larger than 0.95), the Ancc_1_ is always larger than 0.99. Therefore, we can regard 44 as the most proper number of measurements. In the case of azimuth 45° with fewer scattering centers, similar phenomenon can be found and the most proper number of measurements is about 21.

For RV-FSH, the Anccs are lower than those of CV-FSH. The most proper number of measurements is 50 for azimuth 5° and 24 for azimuth 45°, both of which are larger than their counterpart of CV-FSH. These results mean that our CV-FSH outperform RV-FSH on reducing the required number of pulses for HRRP synthesis. This experiment also tells that as long as the number of measurements is sufficiently large, precise HRRPs with slightly decreasing performance can be obtained with much fewer measurements than traditional sense. It is reasonable because the CS-based algorithms perform stably as far as the RIP condition is satisfied, but much worse if the condition is not well satisfied.

**Figure 7.**
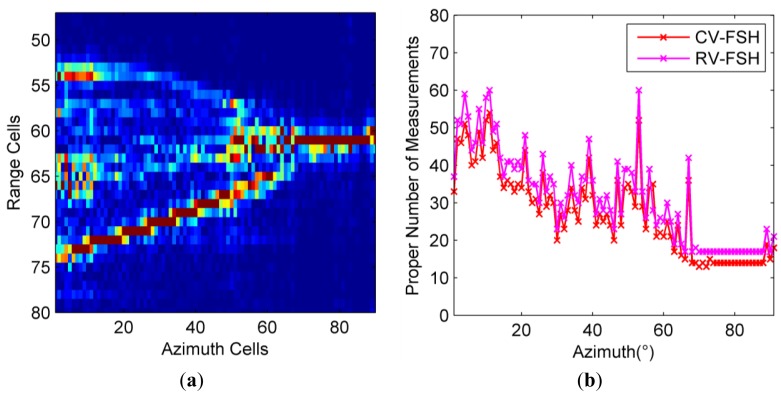
Proper numbers of measurements for HRRP generation. (**a**) Central supports of normalized HRRPs for all azimuths; and (**b**) Proper numbers of measurements for all azimuths.

In order to further validate the effectiveness of the stopping rules, the proper numbers of measurements for generating high quality HRRPs at all azimuths (0° to 90° with an interval of 1°) are calculated. The stopping rules are that the number of measurements is among the presetting scope (from 16 to 80) and the Ancc_1_ is larger than 0.99. The original HRRPs are shown in Figure 7a and the proper numbers are presented in Figure 7b, from which we can see that with the reduction of the number of strong scattering centers projected into the radar LOS, the required number of measurements also decreases correspondingly. Therefore, we can conclude that the number of measurements should be nearly proportional with the number of scattering centers, which is also consistent with the RIP condition. It can be seen again that the required numbers of pulses when resorting to CV-FSH are consistently smaller than that using RV-FSH.

### 5.3. Sequential HRRP Generation of Moving Target

Finally, the performance of velocity measurement and HRRP generation with Algorithm 1 is verified. As the target used for chamber measuring is with no translational motion, we will simulate the echo data by assuming that the target is moving toward the radar with a certain radial velocity *v* and the selected measurements *s_r_*[*m*] are multiplied by the synthesized phase terms corresponding to the radial velocity (*j*π*F_m_*2*vmT_r_*/*c*), *i.e.*:

(40) $$s_{r}^{\prime }[m]=s_{r}[m]\cdot \exp(j2\pi F_{m}2vmT_{r}/c)$$

We still perform the experiments for two typical azimuths (5° and 5°). In order to make the scenario more practical, we assume that the actual radial velocities of the target at azimuth 5° and 45° are respectively 25 m/s and 75 m/s, which is smaller than the maximum unambiguous velocity *c*/(2*f_c_T_r_*) = 150 m/s when the pulse repeat time is 100 us. For the target with higher radial velocity, e.g., 200 m/s, we usually measure its ambiguous velocity as 50 m/s, which belongs to [0 m/s,150 m/s], rather than its true value. Therefore, here we select two typical values (*i.e.*, 25 m/s and 75 m/s) for the target velocities. Assume that the accuracy of tracking system is 5 m/s and measured velocities are 25 m/s and 75 m/s, respectively, then we can regard the velocity domains of interest as [20 m/s,30 m/s] and [70 m/s, 80 m/s]. The velocity domain is divided into *L* = 10 points and then by applying Algorithm 1 to the new data set, we can obtain the velocity estimation as well as the HRRPs.

**Figure 8.**
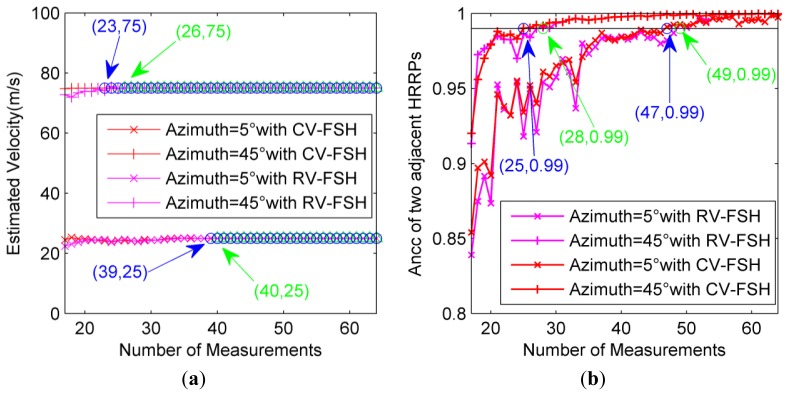
Results of the SCS algorithm for velocity measurement and HRRP generation of the moving target. (**a**) Velocity estimations for various numbers of measurements; and (**b**) Ancc between two adjacent HRRPs.

In order to see the statistic behavior, the experiment is also performed based on 200 Monte Carlo simulations. As shown in Figure 8a, when the number of measurements is larger than 39 for CV-FSH approach or 40 for RV-FSH approach, the velocity estimation of the target at azimuth 5° will be always precise, and for azimuth 45° the minimum number of measurements is about 23 for CV-FSH approach or 26 for RV-FSH approach. Herein, apart from the precise velocity, the Ancc between two adjacent HRRPs should also be large enough, which will finally determine the optimal number of pulses required in each CPI. As shown in Figure 8b, the values of Ancc between two adjacent HRRPs are always larger than 0.99 when the number of measurements exceed 47 for CV-FSH approach or 49 for RV-FSH approach at azimuth 5° and 25 for CV-FSH approach or 28 for RV-FSH approach at azimuth 45°, which are close to the results for the stationary situation in Figure 6. It can be seen again that our CV-FSH algorithm outperforms the RV-FSH algorithm.

## 6. Conclusions

(1) A dynamic compressed sensing strategy for HRRP generation using RSFR is presented in this paper. In this strategy, a complex-valued fast sequential homotopy (CV-FSH) algorithm is proposed to implement the HRRP update quickly with sequentially transmitted and received pulses. By transforming the complex-valued SCS signal model to the group sparse real-valued one *a priori* and adding the real part and imaginary part one by one, CV-FSH performs as an efficient recursive sparse recovery procedure. The stopping rules for this dynamic strategy are based on the one-step agreement of velocity and HRRP. If the sequential estimations of target redial velocity are equivalent and the normalized correlation coefficients between two adjacent HRRPs are sufficiently large, the minimum number of pulses required in each CPI can be determined. The results using simulated and real data show that the proposed algorithm is more suitable for practical application and especially for the uncooperative targets.
(2) Algorithm for fast sequential update is the core of our dynamic compressive sensing strategy. Low computation complexity is the main requirement for this algorithm. It has been demonstrated that our CV-FSH algorithm makes a better tradeoff between reconstruction accuracy and computation complexity, thus it is better for fast sequential update. In the aspect of reconstruction accuracy, simulation results show that CV-FSH is slightly inferior to the algorithm in CVX tool box for precise solution. However, computation load of the CV-FSH is almost 100 times lower than that of the algorithm in CVX tool box under our simulation settings. Moreover, when more and more measurements are available, the computation complexity remains unchanged and even decrease for the CV-FSH algorithm since the results become more and more stable and so fewer iterations are required, while the computation complexity of the algorithm in CVX tool box increases for the larger scale of the sensing matrix. These mean that the algorithm in CVX tool box is not appropriate at all to implement fast sequential update. Compared with RV-FSH algorithm (a direct application of the approach proposed by [14]), CV-FSH algorithm performs better both on reconstruction accuracy and computation complexity. The results using real data show that the required number of pulses when resorting to CV-FSH is reduced consistently by about 10%.
(3) Due to the efficiency and universality, the CV-FSH algorithm can also be used in other CS-based radar applications such as moving target detection [23,24] and cross-range compression in two-dimensional imaging [25,26]. Furthermore, extending the dynamic HRRP generation to the polarimetric case is somewhat straightforward but significant.

## Appendix A

In this appendix, we will give the deviation ∂***x*** of the solutions arising from homotopy parameter increase using the optimality conditions. The optimality conditions are given:

(41) $${\text{A}}_{\Gamma }^{T}(\text{A}{\text{x}}_{k}-\text{y})+{ɛ}_{k}{\text{b}}_{\Gamma }^{T}(\text{b}{\text{x}}_{k}-w)+\lambda \text{D}({\left({\text{x}}_{k}\right)}_{\Gamma })=0$$

(42) $${\text{A}}_{\Gamma }^{T}(\text{A}{{\text{x}}_{k}}^{\prime }-\text{y})+{{ɛ}_{k}}^{\prime }{\text{b}}_{\Gamma }^{T}(\text{b}{{\text{x}}_{k}}^{\prime }-w)+\lambda \text{D}({\left({{\text{x}}_{k}}^{\prime }\right)}_{\Gamma })=0$$

Subtract the left terms of Equation (41) from the left of Equation (42) and make a first-order Taylor approximation to achieve:

(43) $${\text{A}}_{\Gamma }^{T}\text{A}({{\text{x}}_{k}}^{\prime }-{\text{x}}_{k})+{{ɛ}_{k}}^{\prime }{\text{b}}_{\Gamma }^{T}\text{b}({{\text{x}}_{k}}^{\prime }-{\text{x}}_{k})+({{ɛ}_{k}}^{\prime }-{ɛ}_{k}){\text{b}}_{\Gamma }^{T}\text{b}{\text{x}}_{k}+({ɛ}_{k}-{{ɛ}_{k}}^{\prime }){\text{b}}_{\Gamma }^{T}w+\lambda {\text{D}}^{\prime }({\left({\text{x}}_{k}\right)}_{\Gamma })({x_{k}}^{\prime }-{\text{x}}_{k})\approx 0$$

and so the deviation ∂***x*** can be obtained by simplifying the equations in Equation (43).

## Appendix B

In this appendix, the update direction of the solutions and the step size are derived in details. These are separated in the deviation ∂***x*** of the solutions, which is simplified as:

(44) $$\begin{array}{ll} \partial \text{x} & =-\Delta ɛ{\left({\text{A}}_{\Gamma }^{T}{\text{A}}_{\Gamma }+{\text{D}}^{\prime }({\left({\text{x}}_{k}\right)}_{\Gamma })+{{ɛ}_{k}}^{\prime }{\text{b}}_{\Gamma }^{T}{\text{b}}_{\Gamma }\right)}^{-1}{\text{b}}_{\Gamma }^{T}(\text{b}{\text{x}}_{k}-w)\\ & =-\Delta ɛ{\left({\text{A}}_{\Gamma }^{T}{\text{A}}_{\Gamma }+{\text{D}}^{\prime }({\left({\text{x}}_{k}\right)}_{\Gamma })+{ɛ}_{k}{\text{b}}_{\Gamma }^{T}{\text{b}}_{\Gamma }+\Delta ɛ{\text{b}}_{\Gamma }^{T}{\text{b}}_{\Gamma }\right)}^{-1}{\text{b}}_{\Gamma }^{T}(\text{b}{\text{x}}_{k}-w)\\ & =-\Delta ɛ[{\text{U}}^{-1}-{\text{U}}^{-1}{\text{b}}_{\Gamma }^{T}{\left(\frac{1}{\Delta ɛ}+u\right)}^{-1}{\text{b}}_{\Gamma }{\text{U}}^{-1}]{\text{b}}_{\Gamma }^{T}(\text{b}{\text{x}}_{k}-w)\\ & =-\Delta ɛ[{\text{U}}^{-1}{\text{b}}_{\Gamma }^{T}(\text{b}{\text{x}}_{k}-w)-{\text{U}}^{-1}{\text{b}}_{\Gamma }^{T}(\text{b}{\text{x}}_{k}-w)]\\ & =-\Delta ɛ[1-{\left(\frac{1}{\Delta ɛ}+u\right)}^{-1}u]{\text{U}}^{-1}{\text{b}}_{\Gamma }^{T}(\text{b}{\text{x}}_{k}-w)\\ & =\theta_{k}[-{\text{U}}^{-1}{\text{b}}_{\Gamma }^{T}(\text{b}{\text{x}}_{k}-w)] \end{array}$$

Thus the step size is:

(45) $$\theta_{k}=\Delta ɛ[1-{\left(\frac{1}{\Delta ɛ}+u\right)}^{-1}u]=\frac{\Delta ɛ}{1+\Delta ɛu}$$

and the direction is given as:

(46) $$\partial \text{x}=\{\begin{array}{ll} -{\text{U}}^{-1}{\text{b}}_{\Gamma }^{T}(\text{b}x_{k}-w), & \text{on}\;\Gamma \\ 0, & \text{otherwise} \end{array}$$

## Appendix C

In Equation (29), the inactive element *j* is turning into the active one, which means:

(47) $${\text{A}}_{j}^{T}(\text{A}{{\text{x}}_{k}}^{\prime }-\text{y})+{{ɛ}_{k}}^{\prime }{\text{b}}_{j}^{T}(\text{b}{{\text{x}}_{k}}^{\prime }-w)=\lambda \left|\text{D}({\left({{\text{x}}_{k}}^{\prime }\right)}_{j})\right|(47)$$

substituting ${{\text{x}}_{k}}^{\prime }={\text{x}}_{k}+\theta_{k}\partial \text{x}$ into Equation (47):

(48) $$\begin{array}{l} {\text{A}}_{j}^{T}(\text{A}{{\text{x}}_{k}}^{\prime }-\text{y})+{{ɛ}_{k}}^{\prime }{\text{b}}_{j}^{T}(\text{b}{{\text{x}}_{k}}^{\prime }-w)\\ ={\text{A}}_{j}^{T}(\text{A}{\text{x}}_{k}-\text{y})+{ɛ}_{k}{\text{b}}_{j}^{T}(\text{b}{\text{x}}_{k}-w)+\theta_{k}({\text{A}}_{j}^{T}\text{A}+{ɛ}_{k}{\text{b}}_{j}^{T}\text{b})\partial \text{x}+({{ɛ}_{k}}^{\prime }-{ɛ}_{k}){\text{b}}_{j}^{T}(\text{b}{\text{x}}_{k}+\theta_{k}\text{b}\partial \text{x}-w)\\ ={\text{A}}_{j}^{T}(\text{A}{\text{x}}_{k}-\text{y})+{ɛ}_{k}{\text{b}}_{j}^{T}(\text{b}{\text{x}}_{k}-w)+\theta_{k}({\text{A}}_{j}^{T}\text{A}+{ɛ}_{k}{\text{b}}_{j}^{T}\text{b})\partial \text{x}+(\theta_{k}+\theta_{k}\Delta {ɛ}_{k}u){\text{b}}_{j}^{T}(\text{b}{\text{x}}_{k}+\theta_{k}\text{b}\partial \text{x}-w)\\ \approx {\text{A}}_{j}^{T}(\text{A}{\text{x}}_{k}-\text{y})+{ɛ}_{k}{\text{b}}_{j}^{T}(\text{b}{\text{x}}_{k}-w)+\theta_{k}({\text{A}}_{j}^{T}\text{A}+{ɛ}_{k}{\text{b}}_{j}^{T}\text{b})\partial \text{x}+\theta_{k}{\text{b}}_{j}^{T}(\text{b}{\text{x}}_{k}-w) \end{array}$$

where Δε*_k_* =(ε*_k_*′ −ε*_k_*). Since Δε*_k_* ≪ 1 and θ*_k_* < Δε*_k_*, the approximation is reasonable. Thus, the approximation in Equation (32) holds.

## Acronyms

HRRP: High range resolution profile
CV-FSH: Complex-valued fast sequential homotopy
RV-FSH: Real-valued FSH
CPI: Coherent processing interval
PRF: Pulse repetition frequency
PRI: Pulse repetition interval
LSFR: Linear stepped-frequency radar
SSFR: Sparse stepped-frequency radar
RSFR: Random stepped-frequency radar
CS: Compressed sensing
RIP: Restricted isometry property
SCS: Sequential compressed sensing
SNR: Signal-to-noise ratio
T/R: Transmission and reception
BPDN: Basis pursuit denoising
LOS: Line of sight
CVX: Convex

## References

[1] Mensa D.L. (1981). High Resolution Radar Imaging.

[2] Freedman A., Bose R., Steinberg B.D. (1996). Thinned stepped frequency waveforms to furnish existing radars with imaging capability. IEEE Aerosp. Electron. Syst. Mag..

[3] Bose R., Freedman A., Steinberg B.D. (2002). Sequence CLEAN: A modified deconvolution technique for microwave images of contiguous targets. IEEE Trans. Aerosp. Electron. Syst.

[4] Mallat S., Zhang Z. (1993). Matching pursuit with time-frequency dictionary. IEEE Trans. Signal Process..

[5] Tropp J.A., Wright S.J. (2010). Computational methods for sparse solution of linear inverse problems. Proc. IEEE.

[6] Donoho D.L. (2006). Compressed sensing. IEEE Trans. Inf. Theory.

[7] Candès E.J., Romberg J., Tao T. (2006). Robust uncertainty principles: Exact signal reconstruction from highly incomplete frequency information. IEEE Trans. Inf. Theory.

[8] Zhang L., Qiao Z.J., Xing M.D., Li Y.C., Bao Z. (2011). High-resolution ISAR imaging with sparse stepped-frequency waveforms. IEEE Trans. Geosci. Remote Sens..

[9] Zhu F., Zhang Q., Lei Q., Luo Y. (2011). Reconstruction of moving target' HRRP using sparse frequency-stepped chirp signal. IEEE Sens. J.

[10] Liu Z., Wei X.Z., Li X. (2013). Low sidelobe robust imaging in random frequency-hopping wideband radar based on compressed sensing. J. Cent South Univ.

[11] Li G., Meng H.D., Xia X.G., Peng Y.N. (2008). Range and velocity estimation of moving targets using multiple stepped-frequency pulse trains. Sensors.

[12] Axelsson S.R.J. (2007). Analysis of random step frequency radar and comparison with experiments. IEEE Trans. Geosci. Remote Sens.

[13] Candes E.J., Wakin W.M. (2008). An introduction to compressive sampling. IEEE Signal Process. Mag..

[14] Asif M.S., Romberg J. (2010). Dynamic updating for *l*_1_ minimization. IEEE J. Sel. Top Signal Process..

[15] Baraniuk R.G., Davenport M., DeVore R., Wakin W.M. (2008). A simple proof of the restricted isometry property for random matrices. Constr. Approx..

[16] Malioutov D.M., Sanghavi S.R., Willsky A.S. (2010). Sequential compressed sensing. IEEE J. Sel. Top. Signal Process..

[17] Donoho D.L., Tsaig Y. (2008). Fast solution of *l*_1_-norm minimization problems when the solution may be sparse. IEEE Trans. Inf. Theory.

[18] Ender J.H.G. (2010). Oncompressive sensing applied to radar. Signal Process..

[19] Baraniuk R.G., Cevher V., Duarte M.F., Hegde C. (2010). Model-based compressive sensing. IEEE Trans. Inf. Theory.

[20] Lv X.L., Bi G., Wan C. (2011). The group lasso for stable recovery of block-sparse signal representations. IEEE Trans. Signal Process..

[21] Li H.J., Yang S.H. (1993). Using range profiles as feature vectors to identify aerospace objects. IEEE Trans. Antennas Propag..

[22] Grant M., Boyd S. CVX: Matlab Software for Disciplined Convex Programming. http://stanford.edu/~boyd/cvx.

[23] Quan Y.H., Zhang L., Xing M.D., Bao Z. (2011). Velocity ambiguity resolving for moving target indication by compressed sensing. Electron. Lett..

[24] Liu Z., Wei X.Z., Li X. (2013). Aliasing-free moving target detection in random pulse repetition interval radar based on compressed sensing. IEEE Sens. J..

[25] Zhang L., Xing M.D., Qiu C.W., Li J., Sheng J.L., Li Y.C., Bao Z. (2010). Resolution enhancement for inversed synthetic aperture radar imaging under low SNR via improved compressive sensing. IEEE Trans. Geosci. Remote Sens..

[26] Alonso M.T., López-Dekker P., Mallorquí J.J. (2010). A novel strategy for radar imaging based on compressive sensing. IEEE Trans. Geosci. Remote Sens..

